# Interactive technologies in stroke recovery: uncovering challenges and opportunities through physiotherapist’s perspective

**DOI:** 10.1080/07853890.2021.1893582

**Published:** 2021-09-28

**Authors:** S. Pintoa, H. Nicolau, D. S. Lopes, A. M. Pereira

**Affiliations:** a Department of Physiotherapy, Escola Superior de Saúde Egas Moniz , Monte da Caparica , Portugal ;; b Centro de investigação interdisciplinar Egas Moniz , Monte da Caparica , Portugal ;; c Hospital Garcia de Orta , Almada , Portugal ;; d INESC-ID, Instituto Superior Técnico, Universidade de Lisboa , Lisbon , Portugal

## Abstract

**Introduction:**

It is estimated that 55% to 75% of individuals who experience a stroke have persistent impairment of the affected upper limb (UL) [1,2]. It is needed to identify training strategies allied with interactive systems for retraining motor function of the UL. Virtual reality (VR), using either immersive or nonimmersive technology, seems to be one of those promising strategies. Virtual reality allows patients to have close-to-reality experiences, providing them varied, engaging, and realistic experiences [3]. For the physiotherapist, the use of the interactive technologies is a challenge which can improve treatment adherence, allow new environments adapted to patient needs, abilities and goals, as well as different task options [4]. The objective of this analysis was to systematically review the benefits and limitations of VR towards motor recovery of upper limb in post-stroke population.

**Materials and methods:**

Randomised controlled trials were researched in Pubmed and PEDro databases, between January 2009 and January 2019, using the following keywords: “Virtual reality”, “video games”, “upper limb” and “stroke”. We included articles that used immersive and nonimmersive technology in upper limb recovery after stroke, and which compared VR with others modalities We excluded all articles in which the patient received home based intervention or community rehabilitation programs. All included clinical trials had level of evidence equal or superior to 6 score, assessed by PEDro scale.

**Results:**

Fifteen studies met the inclusion criteria. Only three studies considered immersive VR. The training of functional tasks appears to provide the greatest benefits in upper extremity function with improvements in joint range of motion, hand motor function, grip strength, and dexterity. Two studies indicated that long-term improvements persist at follow-up. None of the studies reported any significant adverse effects.

**Discussion and Conclusions:**

There is moderate to high evidence that supports the beneficial effects of VR on stroke patient upper limb motor recovery. However, more studies are needed to determine what kind of VR systems are the most appropriate, particularly which ones may contribute or affect cortical reorganisation. It is also needed to identify the most adequate frequency, duration and intensity for the sessions.
